# The CD19-Coupled CD21 is an Essential Receptor for B cell Activation

**Published:** 2026

**Authors:** Gongyi Zhang

**Affiliations:** 1 Department of Immunology and Genomic Medicine, National Jewish Health, Denver, CO 80206, USA; 2 Department of Immunology and Microbiology, School of Medicine, University of Colorado Anschutz Medical Campus, Aurora, CO 80216, USA

**Keywords:** B cell antigen, CD19, CD21, CD22, C3d, CD45, BCR, CD79A, CD79B, Lyn, Syk, PI3K, LAT2

## Abstract

It has been almost six decades since T cells and B cells were found to be the major players of adaptive immunity. The signal transduction cascades of T cell activation are well studied. However, it is not well appreciated or investigated how the binding of antigens to B cell receptors triggers the downstream signal transduction pathways. It is clear that MHCs with linear antigens presented by antigen-presenting cells (APCs), binding to TCR coupled with CD4/8, is enough to trigger the activation of T cells. For B cells, there are structures of BCRs similar to those of TCRs; however, there are no definitive structures similar to CD4/8 in B cells to help B cell activation. It remains a mystery how B cells are activated by native forms of antigens. Encouraged by our new illustration of the underlying mechanism of T cell activation, a simple model of B cell activation is derived: the CD19-coupled CD21 is an essential receptor for B cell activation.

## Introduction

Compared to the growth factor-driven tyrosine receptors, T cell receptors do not contain any tyrosine kinase motifs in their intracellular or cytosolic regions. However, like cytokine receptors, the intracellular motifs of T cell receptors could recruit non-receptor tyrosine kinases after ligation of their extracellular domains by cognate ligands. Interestingly, the two major ligand-binding receptors of the TCR, TCR-alpha and TCR-beta, do not contain intracellular domains. Instead, there are six additional receptor members, gamma, delta, two epsilon, and two zeta subunits, which contain intracellular motifs but no extracellular motifs to bring non-receptor tyrosine kinases for downstream signaling. Based on two recent high-resolution structural reports of TCRs with and without ligation, which showed no significant conformational changes [1,2], and our reinvestigation of the roles of CD28 [3], we propose a new model of T cell activation (Zhang, under review). Interestingly, there is a similar composition of BCR subunits as that of TCR subunits, and rich biochemical data showed that there must exist a similar signal transduction mode in B cells [4], but it remains not well addressed. Here, we are trying to build up a simple model of B cell activation, which is similar to that of T cells.

## B cell Signaling Model

Compared to T cell receptors, there are similar structures of B cell receptors, which contain two Ig family members with no intracellular structures and two smaller subunits, CD79A and CD79B, with only intracellular domains but without extracellular domains [4]. It is well-characterized that CD79A and CD79B are similar to TCR CD3 zeta subunits, which are essential for intracellular activation and downstream signal transduction, and can be phosphorylated by the SFK member Lyn. The phosphorylation of CD79A and CD79B by Lyn confers their ability to recruit ZAP70-like kinase Syk. It is still not well appreciated that there exists a similar adaptor protein, LAT2 (similar to LAT in T cells) or NTAL (Non-T cell activation linker) in B cells [5]. Furthermore, it is well known that the recruitment of Syk will trigger complete activation of B cells just like T cells. It is also well established that CD19 plays a similar role to CD4 and CD8 in T cells to recruit Lyn to the proximity of CD79A and CD79B in B cells [6]. Interestingly, it was found that CD19 participates in B cell activation coupled with CD21 (CR2, a receptor of C1 complement) [7]. However, the importance of the recruitment of CD21 coupled with CD19 during B cell activation is not well appreciated. People found that the complement system participates in adaptive immunity a long time ago [8]. Many groundbreaking results have been made by Dr. Michael Carroll’s group and others since then [9]. The lectin pathway will trigger the production of C3b after binding to foreign antigens; C3b could be further cleaved to generate C3d, which could directly tag foreign antigens [10,11]. An interesting discovery by Dr. Taras Lyubchenko and his colleagues showed that the binding between CD21 and C3d not only lowers the threshold for B cell activation but also enhances the activation of B cells [12]. From these data and the T cell activation mechanism (Zhang, under review), the B cell activation model could be derived. The binding of C3d-decorated foreign antigen to BCR (usually IgM family members) and CD21 (via C3d) will also bring CD19-coupled CD21 close to CD79A and CD79B (Figure 1A), similar to that of MHCs with antigen binding to TCR coupled with CD4 or CD8. The associated CD19, which is constitutively associated with Lyn [13] (similar to CD4/8 and LCK), will bring Lyn into proximity to CD79A and CD79B. Phosphorylation of CD79A and CD79B by Lyn will recruit Syk. Syk could phosphorylate LAT-like adaptor protein LAT2 or NTAL [5], to trigger the downstream signal cascades, which will lead to B cell clonal expansion in the B cell zone, and also a possible class-switch if they are in the germinal center and differentiate into memory and plasma cells [14] (Figure 1A). In our model, the interaction between CD21 and C3d on the antigen is essential to trigger the activation of B cells, besides the binding of the antigen and BCR, a similar two-component SFK recruiting model of T cell activation (Figure 1A), which is different from the current B cell activation model that the interaction between BCR and antigens will trigger the activation of B cells. From our two-component SFK recruiting model, BCR clustering *via* the binding of antigens alone is not enough to trigger the activation of B cells since it lacks the most critical player, the Lyn-coupled CD19. Lyn, a Src family member (SFK), can only be brought close to the cognate substrates, CD79A/B, by the engagement between CD21 and C3d-decorated antigen, which also binds to BCR through Ig domains of IgM (Figure 1A).

## CD22 in B cells Plays Similar Roles as CD28 in T cells

From the above B cell activation model, we found that CD21 and CD19 work together to act in a similar role to CD4 or CD8 in T cells (Zhang, under review). Interestingly, it was reported that an anti-CD19 monoclonal antibody alone could turn off active B cells [15], reminding us that an anti-CD4 antibody alone leads to the internalization of CD4 and depletion of CD4 T cells [16], further supporting the similar role of CD19 in B cells to CD4 and CD8 in T cells. A question arising is whether there exists a receptor similar to CD28 of T cells in B cells. An early report showed that the addition of an anti-CD22 antibody and an anti-IgM antibody could trigger the proliferation and activation of B cells *in vitro* [17]; this assay reminded us of the famous similar experiments of anti-CD28 antibody and anti-CD3 antibody in the activation of T cells [18,19]. More interestingly, the binding of an anti-CD22 antibody to CD22 not only activated B cells but also coupled with tyrosine phosphorylation of the intracellular motif of CD22 as well as recruitment of Lyn, Syk, and PI3K [20], suggesting CD22 is capable of recruiting Lyn and PI3K in resting B cells, similar to that of CD28 in resting T cells. Other reports also showed that CD22 is the substrate of Lyn [21,22], though people have assigned CD22 as an inhibitory receptor to recruit SHIP in the immunology field [23]. Based on the above analysis, we may speculate that CD22 and its homolog Siglec-G in B cells are likely to act as CD28 in T cells (Figure 1B). This also suggests that anti-CD22 antibody and anti-BCR antibody could activate B cells bypassing CD19, similar to how anti-CD28 and anti-TCR (CD3) antibody could activate T cells bypassing CD4/8.

## CD45 is Associated with CD22 in B cells

CD45 is also expressed in all B cells (CD45 is expressed in all nucleated hematopoietic cells). Interestingly, the knockout of CD45 alone did not show a significant phenotype in B cells. However, double knockout of CD45 and CD148, a partially redundant tyrosine phosphatase, led to a very severe early block in B-cell development [24]. More interestingly, both CD45 and CD22 or Siglec-G are highly decorated with sialic acids and associated with Galectin-9 [23]. These data suggest that CD45 may work together with CD22 or Siglec-G to recruit Lyn and PI3K to keep B cells surviving in normal situations (in resting form), just like what CD45 and CD28 do in T cells [3] (Figure 1B).

## The Underlying Mechanism of B Cell Anergy

It was reported that B cells, like T cells, also go through a negative selection process after birth in bone marrow, which eliminates the high-affinity self-binding IgM population before migrating to the circulation and local lymph nodes [25,26]. People have also proposed a model of self-reactive B cells in circulation or periphery becoming anergic [7]. Based on our above analysis, we may find an alternative interpretation of how high-affinity self-antigen B cells become anergic. Currently, people believe that the interaction between antigens and BCR alone, which will trigger the aggregation of BCRs, will be enough to trigger B cell activation. In this regard, there must exist a mechanism to stop the activation process since lots of self-antigens could bind to the BCRs of B cells with high affinity, so the anergy theory was introduced to solve the self-antigen binding conundrum of IgMs or IgDs. However, in our activation model, when IgM-expressing naïve B cells come across high-affinity self-antigens in the periphery, high-affinity binding between BCR and antigen is not enough to trigger the activation of B cells, since CD79A and CD79B alone, without Lyn, could not trigger activation in our model. There is an essential requirement for the recruitment of Lyn kinase to the proximity of CD79A and CD79B to activate the signal cascade. The binding between CD19-coupled CD21 and C3d becomes essential, which is critical to recruiting Lyn and, consequently, Syk for full activation of B cells (Figure 1A). Surprisingly, an early report from Drs. Lyubchenko and Cambier showed that the signal of CD21 coupled with C3d completely broke the anergy of B cells [27]. The data further support our two-component Src family kinase recruiting (SFK) activation model of B cells. The component of CD19-coupled CD21 and C3d is the second essential component of the model. In this regard, B cells will remain quiescent even when coming across high-affinity antigens since C3d only tags foreign antigens but not self-antigens. These results raise two fundamental questions: Is B-cell negative selection necessary during B-cell development to generate tolerance in animals? Does B-cell anergy exist in animals?

## Conclusion

In conclusion, B cell activation is similar to that of T cells, which require bringing two critical components together: the intracellular portions of CD79A and CD79b *via* the interaction between BCR and antigen, and the corresponding Src kinase Lyn via the interaction of CD19 coupled CD21 and C3d-decorated antigen (Figure 1A). At the same time, naïve B cells can survive in normal situations with CD45 and CD22, which function similarly to CD45 and CD28 in T cells. Furthermore, based on our analysis of CD45 working with CD28 in T cells and working with CD22 in B cells, we speculate that there should exist homologs of CD28 and CD22 in other nucleated hematopoietic cells to maintain the life of cognate cells (Figure 1C). Recently, we proposed that B cells are essential for the development of memory T cells in the germinal centers [14]. At the same time, we also proposed that two-step activation of T cells is essential for the development of long-lived memory T cells (both CD4 T memory cells and CD8 T memory cells) [14], as well as regulatory T cells (Zhang & Li, in press). We believe that it should also be true for memory B cells and Plasma cells. B cells are first primed in B cell zones by C3d-decoreated foreign antigens, while B cells will be activated again by antigens (native form with C3d-decoration) presented by follicular dendritic cells within the germinal centers [14]. With the help of follicular CD4 T cells [14], B cells will go through the maturation process in the germinal centers to become memory B cells and plasma cells.

## Figures and Tables

**Figure 1.**
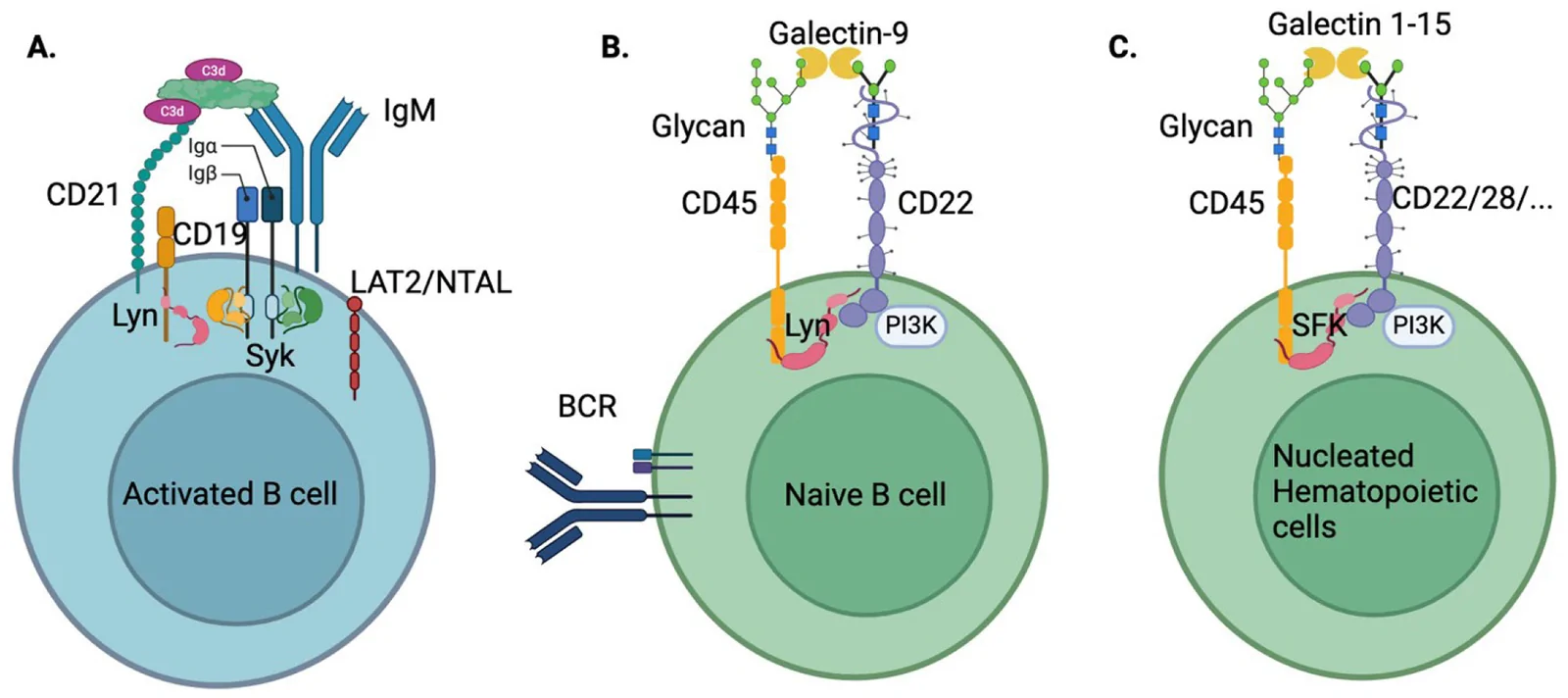
Models of B cell activation and survival. **A.** B cell activation by a C3d-decorated antigen. **B.** Naive B cell is maintained by CD45 and CD22. **C.** CD45 is required for the survival of all nucleated hematopoietic cells.
